# Nursing career frameworks to support career progression: A scoping review

**DOI:** 10.1016/j.ijnss.2026.08.002

**Published:** 2026-09-09

**Authors:** Siobhan Hooper, Craig Phillips, Elizabeth Halcomb, Swapnali Gazula, Hila Dafny, Penelope Wilson, Samantha Jakimowicz, Judith Smith, Peta Hewitt, Jason Mills

**Affiliations:** a Australian College of Nursing, ACT, Australia; b College of Nursing and Health Sciences, Flinders University, SA, Australia; c University of Wollongong, NSW, Australia; d Victoria University, VIC, Australia; e School of Nursing and Midwifery, Adelaide University, SA, Australia; f Charles Sturt University, NSW, Australia

**Keywords:** Career mobility, Nursing, Workforce, Scoping review

## Abstract

**Objective:**

This review aimed to identify, examine, and synthesize current literature on global nursing career frameworks and identify key attributes that effectively support nurses’ career progression.

**Methods:**

A scoping review guided by Arksey and O’Malley’s framework and Joanna Briggs Institute (JBI) best practice guidance for protocol development and reporting. A comprehensive search was conducted across MEDLINE (Ovid), CINAHL (EBSCOhost), Scopus, APA PsycINFO (Ovid), grey literature, and websites of key professional nursing organisations. Sources were included if they were reported in English and focused on the nursing profession and career progression, structured career planning, or career pathway frameworks. Data were synthesized using thematic analysis.

**Results:**

From the 1,159 identified, 12 sources were included. Key attributes of nursing career frameworks that support career progression were identified, including self-directed career planning, clear pathways and role definition, strong leadership and collaborative networks, workforce issues, and prioritizing nurse well-being.

**Conclusion:**

Nursing career frameworks appear to offer strategies that complement workforce recruitment and retention. Such frameworks can provide structured guidance, promote diverse career opportunities, and foster a sustainable nursing workforce.

## What is known?

• The nursing profession faces unprecedented workforce challenges with projections of significant future shortages.
• Nursing career frameworks provide infrastructure to support and develop the capability and capacity of nurses, resulting in transferability across the profession.
• Current nursing career frameworks in countries such as Australia are limited to specialty areas of practice or geographical location.

## What is new?

• Despite evidence to suggest nursing career frameworks can improve retention and attrition rates, there is no overarching framework to guide nurses in countries such as Australia.
• Such frameworks can provide structured guidance, promote diverse career opportunities, and foster a sustainable nursing workforce.
• Nursing career frameworks may effectively complement workforce recruitment and retention strategies.

## 1 Introduction

The global healthcare landscape is currently experiencing unprecedented change and challenges, exacerbated by aging populations, increased patient complexity, rising incidence of chronic disease multimorbidity, population growth, and rapid technological advancements [[1], [2], [3], [4], [5], [6]]. Significant global and local impacts have arisen in this context, resulting in nursing workforce shortages and making the development and implementation of effective, sustainable workforce strategies a critical priority.

As the largest clinical discipline, comprising over 55 % of the healthcare workforce [7], nurses are particularly vulnerable to these challenges. Nurses are uniquely positioned to provide patient-centered care; promote health prevention; educate patients and communities; provide clinical leadership; and advocate for policy change that improves health outcomes and systems [8]. The immense pressure on nurses within the healthcare workforce has led to recruitment challenges, which, in turn, have resulted in workforce shortages, burnout, and decreased job satisfaction [4,5,7,[9], [10], [11], [12]]. Furthermore, due to negative public perceptions of nursing, fewer young people are choosing nursing as a career [3,[13], [14], [15], [16], [17]]. If these issues remain unaddressed, it is predicted Australia will face a shortfall of 70,707 full-time nurses by 2035 [18]. Globally, there is an expected shortage of 4.1 million nurses by 2030 [3,[13], [14], [15], [16], [17]].

Nursing career frameworks serve as critical infrastructure for addressing these workforce shortages by improving employment opportunities, building nurses’ capacity and capability, and facilitating the transferability of nursing skills across diverse healthcare settings [13]. Nursing career frameworks go beyond simply outlining potential career progression to foster improvement in personal and professional identity, increased job satisfaction and higher retention rates [13,[19], [20], [21], [22]]. Additionally, nursing career frameworks can articulate the breadth of the nursing profession, highlight the diverse opportunities available to nurses, and enable nurses to work within the optimal scope of practice [13].

To address critical nursing workforce challenges, one proposal is to develop and implement a nursing career framework to strengthen the nursing profession and ensure a sustainable nursing workforce [4,5,10,13,17,21,23]. Nurses with access to a structured career framework may experience greater recognition within the healthcare workforce, stronger professional and personal identity, and greater career satisfaction. Consequently, providing a structured career framework will increase nurse retention [[21], [22], [23], [24], [25]]. Nursing career frameworks are paramount for enhancing an individual nurse’s professional growth, thereby fostering a diverse and skilled nursing workforce that can optimize patient outcomes [4,[13], [14], [15],^17^,^21^,^25^].

Whilst there are existing career frameworks within the nursing profession [13,[26], [27], [28]], an overarching framework appears to be lacking in countries such as Australia to support all nurses with their career progression, regardless of their location or specialty. This scoping review aimed to identify, examine, and critically synthesize the current literature on nursing career frameworks and identify key attributes to support nurses’ career progression. The research question was “What is known about a nursing career framework in the literature, and what are the key attributes of a nursing career framework in supporting nurses’ career progression?”

This review synthesizes insights from the literature to highlight common themes and foundational elements across nursing career frameworks. Results from this review will contribute to the development of a nursing pathways national career toolkit by the Australian College of Nursing.

## 2 Methods

### 2.1 Review design

A scoping review was undertaken to explore the relevant literature in a specific research area. In line with best-practice guidance for protocol development and reporting, a review protocol was developed and stored on the Open Science Framework (OSF) registry platform *a priori* (https://osf.io/gyzx4/overview). Drawing from Arksey and O’Malley’s methodological framework [29], this scoping review implemented five stages: 1)identification of the research question; 2) identification of relevant studies; 3) selection of eligible studies; 4) charting the data; and 5) collating, summarising and reporting the results.

### 2.2 Literature search

The PICo framework was used to guide and structure the search, comprising a specific Population, phenomenon of Interest (experience or process), and Context: resulting in the following review question: “What is known about nursing career frameworks and their key attributes (I), in supporting career progression (Co) for nurses’ (P)?”.

An initial rapid review was conducted from October 2024 to January 2025 to identify gaps in the literature, keywords, and index terms. The findings from this initial review informed keywords used to develop a complete search strategy for the scoping review. This was followed by a second search in April 2025 using all previously identified keywords and index terms. The databases examined were MEDLINE (Ovid), CINAHL (EBSCOhost), Scopus, and APA PsycINFO (Ovid). Grey literature was identified from: Google and Google Scholar, Grey Source Index, and searches of global professional nursing peak bodies. The initial search sought sources published from 2015 to April 2025. Because most of the research was conducted outside this period, the search dates were extended to 2007–2025. This time period was selected to accommodate internationally developed frameworks. Initially, the search was restricted to the Australian context; however, given the limited scope of the inclusion criteria, international studies were also included. Following the search, all included sources were uploaded to Covidence. Appendix A lists the search strings used for each database.

(1) Inclusion criteria. Sources were included if they were reported in the English language and focused on a) the nursing profession: That is,baccalaureate-prepared registered nurses, diploma-prepared enrolled nurses, and advanced practice nurses working in any setting; b) career progression, structured career planning, or career pathway frameworks or key elements, structures, or strategies for developing a nursing career pathway framework and toolkits; c) any setting where nurses/the nursing profession were engaged was not represented.
(2) Exclusion criteria. Sources were excluded if they focused on a) competency development or competencies required for a specialty area of practice; b) professional development programs; c) reasons for choosing a speciality area or programs to promote choice of specialty area; d) specific career time points.

### 2.3 Data abstraction

Data were extracted into a summary table developed by S. Hooper and reviewed by the other reviewers completing data extraction. The data summary table was developed using Excel and included details about the populations, context, geographical location, study methods, and the phenomenon of interest relevant to the review objective. The reviewers had agreed *a priori* on a standardized process for data extraction that provided a detailed record of decisions regarding the study data and the construction of the data set for synthesis. A third reviewer resolved any conflicts between reviewers at each stage of the screening process.

### 2.4 Narrative synthesis

Data were synthesized using thematic analysis [30]. This method supported a systematic and transparent process of interpretation for narrative synthesis. A summary table was developed to ensure a standardized approach for data extraction, familiarisation, and data analysis. The data extracted for tabulation included key variables such as title/author/year/source, country of origin, study context, sample size, research methods, summary of toolkit, and impact. Four authors independently analyzed the data using the summary table. Themes were discussed and identified between the four authors. Any disagreements were resolved through discussion with the authorship team. In line with the scoping review methodology, quality appraisal, which is routinely undertaken for systematic reviews, was considered outside the scope of requirements for this review [31].

## 3 Results

### 3.1 Search results

The search strategy identified 1,159 sources, all of which were imported into Covidence. Of these, 395 were identified as duplicates, leaving 764 sources. The titles and abstracts were screened independently by two reviewers against the inclusion and exclusion criteria. A further 737 sources were excluded, leaving 27 sources. These were assessed for full-text eligibility independently by four reviewers, and a further 15 sources were excluded. This left 12 sources included in the review, as shown in the PRISMA diagram (Fig. 1). The 12 included sources [4,5,[13], [14], [15], [16], [17],[32], [33], [34], [35], [36]]were from multiple countries including the United Kingdom (*n* = 4), Canada and the United States of America (*n* = 4), New Zealand (*n* = 1), Australia (*n* = 2) and International (*n* = 1). A total of ten journal articles and two website sources were included in the review. It should be noted that all the countries of origin of the included sources have distinct healthcare systems and cultural/clinical practice contexts that naturally inform their respective nursing career development frameworks. As diverse individual-level characteristics are not amenable to thematic organization in narrative synthesis, any reported characteristics relevant to the study aim are presented in tabular form for reference; see Appendix B for details and characteristics of the included studies.

**Fig. 1:**
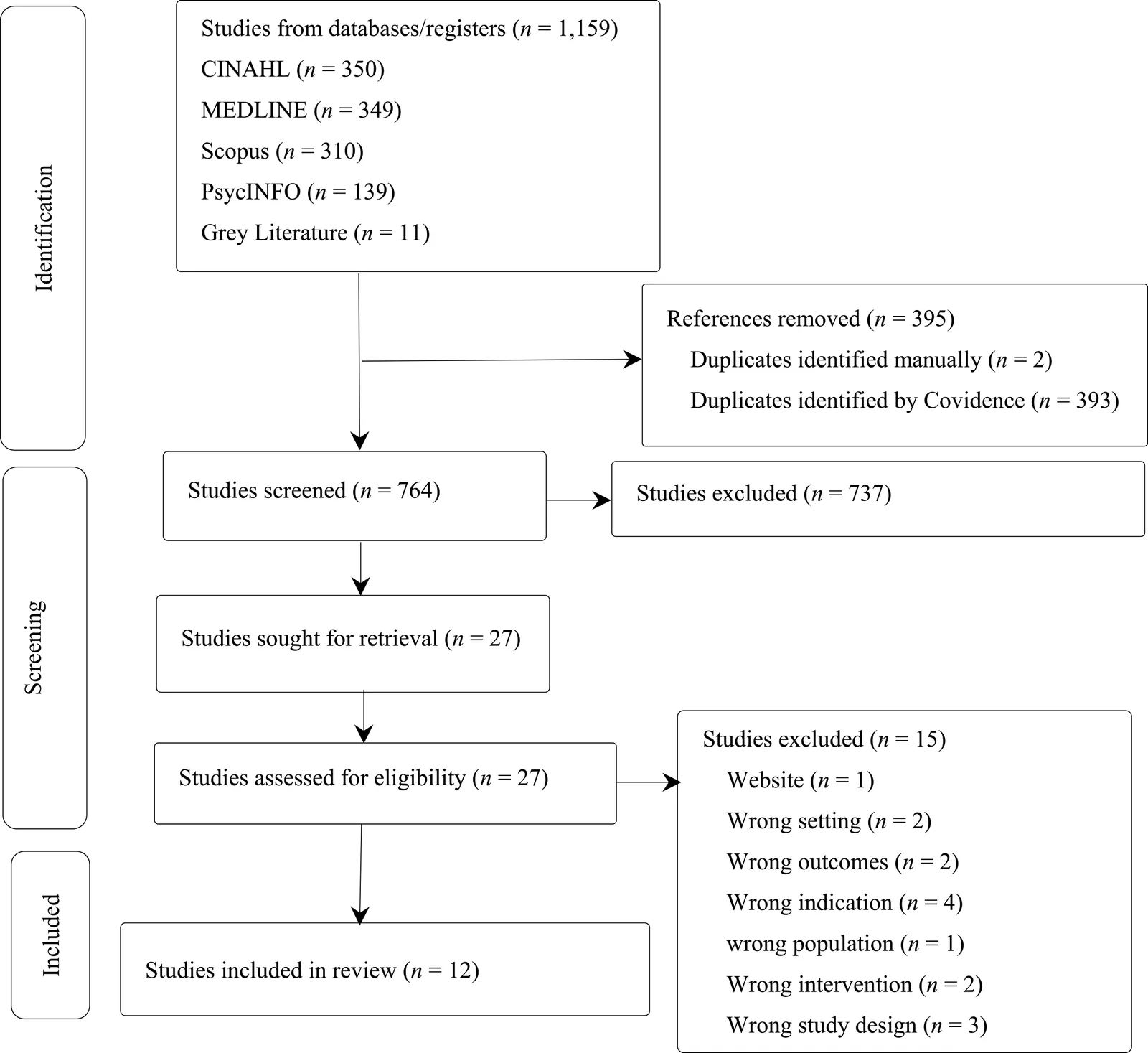
PRISMA flow diagram.

Five themes were identified from the included sources: 1) fostering self-directed career planning; 2) clear pathways and role definition; 3) strong leadership and collaborative networks; 4) workforce recruitment, retention, and capability; and 5) nurse well-being and improving career longevity. Each theme is described in detail below.

### 3.2 Fostering self-directed career planning

Nursing career frameworks focus on empowering nurses to engage in self-directed activities and assessment [32] actively. Self-directed activities include self-assessment, reflection and goal setting, all of which should be fostered and encouraged. Within the nursing profession, reflection should not be limited to clinical reflection but should be incorporated into career planning. Self-assessment tools enable nurses to reflect on capabilities and level of expertise [13,33,34]. Self-reflection empowers nurses to identify their values, experiences, knowledge, strengths, and limitations, and to seek feedback [32] actively. Understanding of their own experience, knowledge, strengths, and limitations enables nurses to incorporate a sound appreciation of their own value into their career planning.

### 3.3 Clear pathways and role definition

Nursing career frameworks incorporating goal-setting components provide nurses with an opportunity to visualize their career progression. It is recommended that, before developing these goals, nurses scan the healthcare environment [3]. Scanning the national, global, and local healthcare environment enables nurses to develop a comprehensive understanding of the broader healthcare landscape. Developing a broader understanding enables nurses to be better informed about issues and trends, assisting in identifying current and future career opportunities [3]. Goal setting is not limited to early-career nurses; rather, it should be incorporated into all career pathways. Nurse leaders play a critical role in supporting and encouraging nurses to set career goals; however, it is imperative that leaders themselves set their own career goals to continue their professional development [34].

Nursing career frameworks need to reflect contemporary nursing and highlight the diversity of the nursing profession [5,17,32]. Highlighting this contemporary image of today’s nurse helps minimize barriers that nurses in non-clinical or non-traditional nursing roles often experience [3,4,16,33,35]. Defining and providing examples of each pathway, with practical guidance on how to transition into other areas, facilitates career progression and reduces uncertainty when considering alternative pathways [5]. Given the wide variety of specialist nurses within the healthcare system, clearly defined pathways will help address inaccurate perceptions of nurses’ scope of practice [4,5,13,33]. Frameworks which build on transferable skills enable nurses to transition into alternative career pathways [13]. Nursing frameworks should be inclusive, providing a structured guide for nurses within each stage of the pathways [16,17].

### 3.4 Strong leadership and collaborative networks

Nurses’ career progression is significantly supported by strong leadership and access to collaborative networks. Leadership is viewed not only in the context of employment but also as an opportunity to mentor. Mentorship, both formal and informal, is a key strategy for professional development in nursing careers [3,13,15,33,34,36]. Mentorship significantly reduces attrition rates in the nursing profession, supports succession planning, and guides nurses, especially those in non-clinical roles [14,34,36].

The development of nursing career frameworks through collaboration with a diverse range of stakeholders ensures they remain inclusive, responsive, and contextually relevant. Stakeholders may include nursing professionals, educators, policymakers, professional peak bodies, and healthcare organizations. Through collaboration, developers gain a comprehensive understanding of the unique needs, priorities, and challenges experienced by end users, nurses [14,35]. Collaboration is also evident when frameworks build upon and integrate existing frameworks. This approach recognizes prior work, leverages established best practices, and promotes continuity across the nursing workforce landscape [5]. Nursing career frameworks should highlight the importance of seeking out opportunities for nurses to receive financial assistance to undertake professional development activities or to apply for scholarship funding for further education [13,14].

### 3.5 Workforce recruitment, retention, and capability

Nursing career frameworks play an important role in improving nurse recruitment and retention and in developing nurses’ capabilities [5,13,16]. This is especially relevant in nursing specialties and non-clinical roles that require enhancement and recognition of nurses’ capabilities outside clinical settings. For example, improving the recruitment and retention of nurses in research, academic roles, and entrepreneurial roles will be a step towards addressing the imminent nursing workforce crisis [3,4,13,15,33].

Research is a fundamental driving force that ensures the healthcare industry maintains evidence-based practices. Historically, there have been many barriers to nurses applying for and securing research funding [14,15]. Nursing career frameworks that incorporate building nurses’ research capacity and increasing awareness of financial assistance may enable nurses to progress in this area of practice and enhance their research capability. This will, in turn, improve patient outcomes and improve quality of care [13,14].

A nursing career framework that reflects and supports a contemporary, diverse image of nursing is imperative to addressing the recruitment issues currently facing the nursing profession. Nursing career frameworks that demonstrate varied career choices help improve the image of nurses, which is often perceived as inaccurate and inconsistent [3,5,33].

### 3.6 Nurse well-being and improving career longevity

An often overlooked yet critical attribute of a robust framework is the integration of nurse well-being and self-care practices to ensure career sustainability and longevity. Self-care is central to the care of others, career progression and leadership development [16,34]. Self-care strategies are pivotal in reducing burnout, stress, and fatigue experienced by a large proportion of nurses [5,33]. A nursing career framework that acknowledges the challenges nurses face in maintaining a healthy work-life balance enhances career longevity. Recognizing and promoting skills and strategies to enhance work-life balance is crucial for supporting nurses’ career progression [36]. The notion of work-life balance differs among nurses. According to a study conducted by Vannucci and Weinstein, nurse entrepreneurs achieve work-life balance through psychological empowerment rather than self-care practices [33]. Nursing career frameworks that recognize the unique connectedness to self and others allow nurses to navigate challenges to progress within their career proactively [15,34].

## 4 Discussion

This scoping review identified the key attributes of nursing career frameworks that support nurses’ career progression. A well-designed nursing career framework plays a critical role in addressing nursing recruitment, workforce shortages and retention issues [5,13,15,23,[36], [37], [38]]. Additionally, nursing career frameworks are essential to fostering a positive professional and personal identity for nurses, leading to increased job satisfaction [20,22,[39], [40], [41]].

Nursing career frameworks enable nurses to articulate and visualize the innovative roles available within the nursing profession [4,5,14,17,21]. Traditionally, nursing career frameworks have focused on acute clinical settings. Frameworks developed by the Australian Primary Health Care Nurses Association [13] and the Australian framework for cancer nurses [4] alongside pathways for clinical educators [14,16] and entrepreneurial nurses [33], highlight the opportunities for nurses today. The provision of a clear nursing career framework is ‘not merely a way to map out potential progression’ [21]; rather, it provides nurses recognition for the diversity of qualifications, enabling nurses to work to their full scope of practice [42].

A nursing career framework provides nurses with guidance to navigate the often overwhelming and complex nature of the modern nursing profession [43]. Frameworks can empower nurses to proactively invest in their career planning through fostering self-directed learning and environmental scanning [3,34]. Self-directed learning encourages nurses to understand their personal strengths, limitations, and values, and enables them to align their careers with individual interests rather than organizational needs [3].

Equally important to career planning is increasing awareness of the broad healthcare landscape. By developing an understanding of the profession as a whole, nurses can identify global, national, and local trends affecting the profession and, consequently, recognize challenges and opportunities as they arise [3]. By completing self-directed learning and undertaking environmental scans, nurses are empowered to identify and develop their own achievable career goals, thereby increasing opportunities for their career progression [3,34].

Providing and maintaining a supportive environment through nursing career frameworks is essential to ensuring nurse retention throughout their careers. Support should be identified and uniquely addressed within nursing career frameworks, tailored to different pathways [38,44]. Although support is viewed through different mechanisms, mentorship is consistently emphasized across frameworks. Mentorship is a collaborative relationship between a more experienced nurse (mentor) and a less experienced nurse (mentee) [36]. Mentorship is viewed as peer support that enables the mentee to develop professionally, increase their job satisfaction, and helps the mentor create a positive workplace environment [13,36,42]. Additionally, mentorship programs assist nurses transitioning between pathways by fostering a sense of connection and belonging [14,36].

Support is provided by ensuring nurses are recognized regardless of their practice setting. This acknowledgment enables nurses to receive support even in non-traditional nursing roles, such as clinical academics, researchers, and entrepreneurs [14,16,33,35].

Supportive environments are not limited to the physical environment. Financial support should also be considered. Financial support is crucial for nurses pursuing career progression, as a lack of adequate funding is recognized as one of the main barriers to nurses' progression in non-clinical roles [14,16,33,35].

It is paramount that nurses’ well-being is acknowledged in nursing career frameworks for many reasons, particularly to minimize the incidence of compassion fatigue. The caring and compassionate role of nurses has made this profession particularly susceptible to compassion fatigue compared with other healthcare professions [45]. The physical manifestation of compassion fatigue is recognized as exhaustion in all aspects of a person’s health. Compassion fatigue is associated with continuous exposure to others' suffering and/or work stress [45].

Although not explicitly addressed in all nursing career frameworks, aspects related to nurses' well-being are correlated with job satisfaction on the one hand and with burnout, stress, and fatigue on the other [5,23,37,38]. Nursing career frameworks highlight the importance of nurses’ well-being and provide strategies to indirectly support nurses, enhancing their identity and self-efficacy [13,20,23]. Additionally, providing resources within nursing career frameworks for self-care practices and processes to help nurses cope with stress ensures they can sustain their professional engagement. In turn, their ability to manage stress can assist nurses in managing stressful situations and enable them to progress in their careers [33,34,36].

### 4.1 Limitations

The specific countries, cultures, and professional/nursing contexts associated with each of the included sources mean that direct transferability to nurses from other countries and cultures may be limited. Additionally, the diversity of sources included in this review reflects both strengths and potential limitations. The sources range from empirical data derived from primary research studies to published frameworks on professional association websites and within other grey literature. While formal quality appraisal of studies was not required, according to scoping review methodology [31,46], this diversity of the literature represents a study limitation regarding hierarchies of evidence and peer review. Furthermore, the inclusion of grey literature and conceptual papers, whilst enhancing the results, means that not all of the attributes discussed are supported by empirical data [3,5,14,15,36].

The grey literature included in this review provides a strong representation of nursing in Australia and New Zealand whilst also indicating potential geographical or cultural bias [5,13]. While the sample size of studies in this review may be considered a limitation, this is not uncommon for review questions focused on specific areas of nursing, and even empty reviews can be valuable for answering questions in a secondary research context. Alternatively, a modest breadth or depth of literature may be interpreted as reflecting the underdeveloped state of research and evidence base regarding nursing career frameworks that support career progression. On that basis, the findings of this review, along with ongoing appraisal of future research literature, will be important as developments in this area grow.

### 4.2 Implications

The findings of this review offer significant implications for the nursing profession and the global healthcare landscape. These implications are multifaceted, impacting nurses directly, other health professionals, and ultimately, all patients receiving nursing care. Currently, the nursing profession globally faces unprecedented challenges related to retention [42,47]. Nursing career frameworks are powerful tools for addressing retention issues, as they provide nurses with clear pathways that highlight career progression, educational opportunities, and support mechanisms to help them advance [4,5,13,21,23,36,38]. The need for such frameworks is apparent internationally, with career planning and development frameworks recommended from research in countries such as China to address nursing workforce concerns and reduce turnover intention [48,49]. Furthermore, nursing career frameworks produce a highly skilled and diverse nursing workforce, which is crucial for delivering evidence-based practice that enhances patient and health outcomes [4,14,17,21,35,38].

The development and promotion of nursing career frameworks can significantly improve the image of nursing. Articulating the breadth and depth of the nursing profession, especially in non-traditional roles, provides health professionals and the public with a contemporary view of nursing that emphasizes nurses’ expanded scope of practice [4,5,13,16,17,33,35,42].

The findings from this scoping review will inform the development of an overarching nursing career framework. A nursing career framework such as this may assist healthcare organizations and policymakers in developing strategic workforce development strategies. Workforce planning identifies current and future trends and recognizes the transferability of nurses’ skills, thereby providing greater flexibility and responsiveness to the delivery of healthcare [5,13,19,23,38].

### 4.3 Future research

Based on the findings and limitations of this study, several areas for future research emerged. The literature provides substantial evidence of the effectiveness of nursing career frameworks. However, future research is required to evaluate the long-term effectiveness from a nurse's perspective [35]. An in-depth evaluation should explore the effectiveness of specific support mechanisms and their impact on nurse career progression, job satisfaction, and overall retention in the nursing profession. This research highlighted the limited number of nursing career frameworks; therefore, it suggested research to understand the barriers and facilitators to implementation, with the potential to ensure a sustainable nursing career framework that effectively supports nurses globally [14]. The implementation of nursing career frameworks is not limited to experienced s; practitioners; rather, these frameworks have the potential to inform and assist nursing students in their transition to practice as registered nurses, with respect to career/practice setting choice.

## 5 Conclusion

The trajectory of a nurse's career is multifaceted and dynamically influenced by a plethora of elements, including individual career planning, organizational structure and the broader social context. Nursing career frameworks comprising effective career planning and wellbeing strategies, clear pathways, and supportive networks, both organizationally and personally, are crucial for supporting nurses in their career progression. Subsequently, nursing career frameworks address global nursing workforce challenges including recruitment, retention, and shortages. Ultimately, nursing career frameworks highlight the breadth and depth of the nursing profession, offering a contemporary view. As the largest and most widely dispersed healthcare profession, the nursing profession must cultivate a workforce that is effectively supported, diverse and highly skilled. This will contribute to a profession that attracts more new nurses, improves nurse retention, and ultimately achieves better global health outcomes.

## Data availability statement

The datasets generated during and/or analyzed during the current study are available from the corresponding author upon reasonable request.

## CRediT authorship contribution statement

**Siobhan Hooper:** Conceptualization, Funding acquisition, Project administration, Methodology, Investigation, Data curation, Visualization, Formal analysis, Writing – original draft, Writing – review & editing. **Craig Phillips:** Investigation, Data curation, Visualization, Formal analysis, Writing – review & editing. **Elizabeth Halcomb:** Methodology, Investigation, Data curation, Visualization, Formal analysis, Writing – review & editing. **Swapnali Gazula:** Methodology, Investigation, Data curation, Visualization, Formal analysis, Writing – review & editing. **Hila Dafny:** Funding acquisition, Investigation, Data curation, Visualization, Formal analysis, Writing – review & editing. **Penelope Wilson:** Investigation, Data curation, Visualization, Formal analysis, Writing – original draft, Writing – review & editing. **Samantha Jakimowicz:** Investigation, Data curation, Visualization, Formal analysis, Writing – review & editing. **Judith Smith:** Investigation, Data curation, Visualization, Formal analysis, Writing – original draft, Writing – review & editing. **Peta Hewitt:** Conceptualization, Writing – review & editing. **Jason Mills:** Conceptualization, Funding acquisition, Investigation, Data curation, Visualization, Formal analysis, Writing – original draft, Writing – review & editing.

## Funding

The first author acknowledges scholarship funding from the10.13039/100015539Australian Government National Industry PhD (NiPhD) Program award. The funding organization had no other role in the conduct of this study.

## Declaration of competing interest

The authors have declared no conflict of interest.
