# Supplementary material for: Nursing career frameworks to support career progression: A scoping review
Source: Int J Nurs Sci. 2026 Sep 9;13(5):520–6. doi: 10.1016/j.ijnss.2026.08.002 (PMC13648174; doi:10.1016/j.ijnss.2026.08.002)
Supplement: Supplementary file 1 [file mmc1.docx]

| Appendix A. Strategies of literature search | |
| --- | --- |
| Database | Retrieval strategies |
| MEDLINE (Ovid) | #1 nurs*.ti,ab.  #2 exp Nurses/  #3 1 OR 2  #4 (leadership* OR mentor* OR network* OR guidance*OR support* OR coach* OR financ* OR clinical supervis*).ti,ab. #4 Mentors/ OR Leadership/ OR (Mentoring/ OR Preceptorship/) #5 Mentors/ OR Leadership/ OR (Mentoring/ OR Preceptorship/)  #6 4 OR 5  #7 (career progression* OR promotion* OR career advancement* OR career development* OR career trajectories OR career path*).ti,ab.  #8 Career Choice/  #9 7 OR 8  #10 (framework* OR toolkit* OR toolbox* OR "tool kit*" OR "tool box*" OR model* OR guideline* OR strateg*).ti,ab.  #11 3 AND 6 AND 9 AND 10  #12 limit 11 to (English language and yr="2015 -Current") |
| APA PsycInfo (Ovid) | #1 nurs*.ti,ab.  #2 exp Nurses/  #3 1 or 2  #4 (leadership* OR mentor* OR network* OR guidance*OR support* OR coach* OR financ* OR clinical supervis*).ti,ab.  #5 Mentor/ OR exp Leadership/ OR exp Professional Supervision/  #6 4 or 5  #7 (career progression* OR promotion* OR career advancement* OR career development* OR career trajectories OR career path*).ti,ab.  #8 (framework* OR toolkit* OR toolbox* OR "tool kit*" OR "tool box*" OR model* OR guideline* OR strateg*).ti,ab.  #9 3 and 6 and 7 and 8  #10 limit 9 to (english language and yr="2015 - 2025") |
| APA PsycInfo (Ovid) | S1 (MH "Nurses+") OR TI(nurs*)  S2 (MH "Mentorship") OR (MH "Clinical Supervision") OR (MH "Leadership") OR AB (leadership* OR mentor* OR network* OR guidance*OR support* OR coach* OR financ* OR "clinical supervis*")  S3 (MH "Career Planning and Development") OR AB ("career progression*" OR promotion* OR "career advancement*" OR "career development*" OR "career trajectories" OR "career path*")  S4 AB (framework* OR toolkit* OR toolbox* OR "tool kit*" OR "tool box*" OR model* OR guideline* OR strateg*)  S5 S1 AND S2 AND S3 AND S4  S6 S5 limited to 2025-2025, English Language |
| Scopus | #1 ( TITLE-ABS-KEY ( nurs* )  AND  TITLE-ABS-KEY ( leadership* OR mentor* OR network* OR guidance*OR AND  support* OR coach* OR financ* OR "clinical supervis*" )  AND  TITLE-ABS-KEY ( "career progression*" OR promotion* OR "career advancement*" OR "career development*" OR "career trajectories" OR "career path*" ) AND TITLE-ABS-KEY ( framework* OR toolkit* OR toolbox* OR "tool kit*" OR "tool box*" OR model* OR guideline* OR strateg* ) )  AND  PUBYEAR > 2014 AND PUBYEAR < 2026 AND ( LIMIT-TO ( LANGUAGE , “English”) ) |

Appendix B. Data extraction summary table

| Title/author/year/source | Country of study | Target group | Recruitment methods/  study type/ ethics | Study context | Sample size | Data collection method | Data analysis method | Research question/aim | Summary of toolkit including impact | Further research and barriers |
| --- | --- | --- | --- | --- | --- | --- | --- | --- | --- | --- |
| APNA Career and education framework  Australian Primary Health Care Nurses Association [13], 2018)  APNA Website | Australia and NZ | RN in PHC settings | Consultation phase (2016-2017) involving >1,000 stakeholders; Delphi study method; workshops and surveys | Across Australia and NZ | > 1,000 stakeholders | Brainstorming, briefings, Delphi methodology, interviews & workshops. | n/a | Improve employment workplace support transferability of nursing skills across PHC settings. | Self-assessment tool for RNs to reflect of expertise across 5 domains  Support performance reviews, job applications, position descriptions and workforce planning | Linked to My Nursing Future Integration of mentors and career planning.  Limited to PHC nurses and 5 broad domains |
| Developing a CA career pathway in a Community and Mental Health NHS Trust  Bernhardt et al. [14], 2023  Journal of Research in Nursing | UK | Nurses AHPs, healthcare professionals | n/a | Community and Mental Health NHS Trust | Not specified | Case study | Case study analysis | Describe the initial development of a CA career pathway | Academic supervision, research collaboration, flexible study options  Increase research capacity and capability, , research culture, visibility and role modelling improved engagement  Linked to positive patient outcomes | Funding through scholarships and fee waivers, incorporated Kotter’s (1996) Change Model.  Limited funding for nurses, lack of recognition and resources and low CA participation  Specific to CA. |
| Essentials of building a career in nursing research.  Cleary et al. [15], 2015  Nurse Researcher | Australia & UK | Emerging and beginner researchers | Discussion paper | n/a | n/a | Discussion | Discussion | Explores transition and career essentials for nurse researchers. | Networking, funding, mentoring and publications assistance  Research fundamental to evidence-based practice. Development of capabilities and capabilities, mentoring and supervision fundamental to transition success. | Academic qualification alone insufficient preparation for nurse researchers. Funding competitive  Study limited to speciality area |
| Interventions and strategies aimed at clinical academic pathway development for nurses in the United Kingdom: A systematised review of the literature.  Henshall et al. [35], 2021  *Journal of Clinical Nursing* | UK focused | Nurses, midwives and AHP | Systematic review | UK | Ten papers | Literature review (2006 – 2020) | Narrative analysis | Review strategies, innovations and initiatives to promote CA pathways | Core components include Leadership, communication, partnerships and strategic aims.  Increased development of CA pathways, education and funding | Coordinated National response and approach to CA nursing research pathway. Lack of CA pathways. |
| Career Planning and Development. It’s your career: take charge workbook  International Council of Nursing [32], 2010  ICN Website | International | Nurses | n/a | n/a | n/a | n/a |  | Support proactive career planning | Holistic, self directed workbook  Nurses become aware and take responsibility for career planning and development | Requires updating and country specific content |
| Building Career Pathways for Cancer Nurses: Ensuring Cancer Nursing’s Future.  McErlean et al. [4], 2024  *Seminars in Oncology Nursing* | Australia | Cancer nurses and advanced practice nurses | n/a | n/a | n/a | review guided by the Australian Cancer Plan | n/a | Develop evidence-informed cancer nursing career framework | Contemporary career framework with clear pathways incorporating novice to expert model  Reflects contemporary cancer care, to support workforce sustainability and transition into speciality areas. Includes implementation of ACP. | Nationally agreed speciality framework. |
| A Career Framework for the Health Workforce in New Zealand. Wellington: Ministry of Health and District Health Boards New Zealand.  Ministry of Health and District Health Boards New Zealand Workforce Group [5], 2007  Ministry of Health NZ | NZ | Healthcare workforce | Consultative project | NZ Health workforce | n/a | Critical perspective | n/a | Support individualised career planning and workforce development | Framework outlining pathways, transition, to support recruitment and retention ensuring diverse workforce.  Supports recruitment and retention, improve image of HCP. | Ongoing testing and evaluation recommended.  Concerns of NZ nursing shortages not meeting population needs. |
| Florence Nightingale’s legacy for CA: a framework analysis of a clinical professorial network and a model for clinical academia.  Pattison et al. [16], 2020  *Journal of Clinical Nursing* | UK | CA nursing professors | n/a | Northern Ireland, Scotland  and England | n/a | Written templated | Qualitative framework thematically analysed. | Exploration of CA professors roles and impact. | Eight theme identified.  Highlights CA stages and improves image. Facilitates translation of research into clinical practice | n/a |
| CALM^©^: A Customized Advanced Leader Model for Enhancing Career and Well-Being.  Pinekenstein & Pawlak [34], 2020  *Nurse Leader* | USA | Nurse leaders | n/a | n/a | n/a | n/a | n/a | Supports professional growth and wellbeing | Leadership models focusing on personal values, mentoring and self-awareness enhance career progression.  Supports succession, planning to reduce attrition. | There are significant incidence of burnout, fatigue and stress within nursing.  Sel care a priority for nurses. |
| The Exemplary Practice Life of the Nurse.  Riley et al. [17], 2021  Journal of Professional Nursing | USA | RN | n/a | n/a | n/a | n/a | n/a | Describe exemplary nursing practice | Innovative holistic conceptual model exploring scholarly practices, leadership and stewardship  Practical model to translate nursing competences for students and RN’s. | n/a |
| Establishing a mentorship program in rural workplaces: Connection, communication, and support required  Rohatinsky et al. [36], 2020  Rural and Remote Health. | Canada | Rural mentor and mentee s. | Recruitment through health region.  Ethics approval obtained | Saskatchewan province in Canada | 15 mentees and 43 mentors. | Telephone interviews and evaluation survey | Thematic analysis | Exploring mentoring as a recruitment and retention strategy | Mentorship focused on supporting HCP in rural areas through connection, communication Mentorship beneficial to support HCP in rural environments. | Strengthening mentoring relationship requires improvement  Focuses specifically on Canada |
| The nurse entrepreneur: empowerment needs, challenges, and self-care practices.  Vannucci & Weinstein [33], 2017  *Nursing: Research and Reviews* | USA & Canada | Nurse entrepreneurs | Email via professional networks | USA & Canada | 44 participants | Online survey including Likert-type and open-ended questions. | Thematic analysis | Explore experiences and challenges of nurse entrepreneurs | Psychological empowerment primary motivator  Higher rates of self-care for nurses working exclusively in their own business | Explore the self-care and work/life balance experiences of nurse entrepreneurs in comparison to nurses in other employment scenarios  Work/life balance challenges, implementing a marketing strategy,  networking, and accessing mentorship |

*Note*: APNA = Australian Primary Health Care Nurses Association.
