# Supplementary material for: Nursing career frameworks to support career progression: A scoping review
Source: Int J Nurs Sci. 2026 Sep 9;13(5):520–6. doi: 10.1016/j.ijnss.2026.08.002 (PMC13648174; doi:10.1016/j.ijnss.2026.08.002)
Supplement: Supplementary file 2 [file mmc2.docx]

**护理职业发展支持框架研究：一项范围综述**

Siobhan Hooper, Craig Phillips, Elizabeth Halcomb, Swapnali Gazula, Hila Dafny, Penelope Wilson, Samantha Jakimowicz, Judith Smith, Peta Hewitt, Jason Mills

**【摘要】**

**目的** 识别、审查并综合当前关于全球护理职业框架的文献，确定有效支持护士职业发展的关键要素。

**方法** 根据Arksey和O’Malley的框架以及乔安娜・布里格斯研究所关于方案设计与报告的最佳实践指南进行范围审查。计算机检索MEDLINE(Ovid), CINAHL (EBSCO Host), Scopus, APA PsycINFO (Ovid)4个在线数据库、灰色文献及专业护理组织。纳入文献以英语发表，并聚焦于护理职业、职业发展、结构化职业规划或职业路径框架等内容。数据通过主题分析法进行综合整理。

**结果** 共检索1 159篇文献，最终纳入12篇文献。护理职业框架的关键特征包括支持职业发展的要素，如自主的职业规划、清晰的职业发展路径与角色定义、强有力的领导力和协作网络、人力资源问题以及优先关注护士的福祉。

**结论** 护理职业框架提供了有助于补充劳动力招聘与留任的策略、能够提供结构化的指导，促进多样化的就业机会，并推动护理人才的可持续发展。

【**关键词**】职业流动性；护理；人力；范围综述

通信作者：Jason Mills, E-mail: [jason.mills@flinders.edu.au](mailto:jason.mills@flinders.edu.au)
